# Unimpaired Neuropsychological Performance and Enhanced Memory Recall in Patients with Sbma: A Large Sample Comparative Study

**DOI:** 10.1038/s41598-018-32062-5

**Published:** 2018-09-11

**Authors:** S. Marcato, J. R. Kleinbub, G. Querin, E. Pick, I. Martinelli, C. Bertolin, S. Cipolletta, E. Pegoraro, G. Sorarù, A. Palmieri

**Affiliations:** 1 Department of Neurosciences (DNS), University of Padova Via Giustiniani, 2 - 35128 Padova, Italy; 2 Department of Philosophy, Sociology, Pedagogy and Applied Psychology (FISPPA), University of Padova Piazza Capitaniato, 3 - 35139 Padova, Italy; 3 Department of General Psychology, University of Padova via Venezia, 8 -35131 Padova, Italy; 4 Padova Neuroscience Center (PNC), University of Padova Via Orus, 2 - 35129 Padova, Italy

## Abstract

Peculiar cognitive profile of patients with SBMA has been described by fragmented literature. Our retrospective study reports the neuropsychological evaluations of a large cohort of patients in order to contribute towards the understanding of this field. We consider 64 neuropsychological evaluations assessing mnesic, linguistic and executive functions collected from 2013 to 2015 in patients attending at Motor Neuron Disease Centre of University of Padova. The battery consisted in: Digit Span forwards and backwards, Prose Memory test, Phonemic Verbal fluency and Trail making tests. ANCOVA statistics were employed to compare tests scores results with those obtained from a sample of healthy control subjects. Multiple linear regressions were used to study the effect on cognitive performance of CAG-repeat expansion, the degree of androgen insensitivity and their interaction to cognitive performance. Statistical analyses did not reveal altered scores in any neuropsychological tests among those adopted. Interestingly, patients performed significantly better in the Prose Memory test’s score. No relevant associations were found with genetic, hormonal or clinical patients’ profile. Results inconsistent with previous studies have been interpreted according to the phenomenon of somatic mosaicism. We suggest a testosterone-related and the mood state-dependant perspectives as two possible interpretations of the enhanced performances in the Prose Memory test. Further studies employing more datailed tests batteries are encouraged.

## Introduction

X-linked spinal and bulbar muscular atrophy (SBMA), or Kennedy’s Disease (KD), is a rare neurological disorder caused by a CAG-repeat expansion in the first exon of the androgen receptor (AR) gene^1^. This sequence expansion leads to insensitivity to androgens and neurodegeneration mainly at neuromuscular level. Kennedy’s disease usually affects patients between 30 and 50 years of age. Its prevalence is estimated to be 2.8/100.000 in the male population^2,3^. Interestingly, in addition to lower motor neurons in the spinal cord and brainstem, mutant AR accumulation has been observed in a wide array of neural cells of SBMA patients including cortical neurons^4^, which could therefore result in cognitive alterations.

Neuroimaging and electrophysiological studies as well indicate contrasting results, reporting different levels of cerebral involvement other than primary motor cortex, in which it can be expected^5–12^. In detail, studies employing MRI reported atrophy of white matter mainly in frontal areas – but also in subcortical ones, limbic structures, cerebellum, and brainstem – suggesting, as a whole, multifocal white matter atrophy and possible grey matter loss in frontal areas^6,8,11,12^.

Briefly, although these findings mostly seem to suggest a possible hypometabolism of the frontal lobes, the morpho-functional involvement of the central nervous system of these patients is still not clear, hence a straightforward direction to investigate any specific cognitive deficit is hard to be traced.

However, these patients’ peculiar cognitive profile, if present, is still not clear, as few studies are available in literature on this topic and, because of the rarity of the disease, they are based on small sample sizes. In detail, heterogeneous results in their neuropsychological profile have been described, and different deficits – from subclinical to frank level – in short- and long-term memory, attentional control, executive functions or ToM abilities have been found^13–17^. Sporadic cases of association between SBMA and frontal type dementia have also been reported^18,19^.

In this vein, Finsterer and Sorarù^3^, in their recent review on primary and secondary symptoms of patients with SBMA, explicitly refer to their eventual cognitive impairment as a “questionable manifestation”.

Given this fragmented panorama, the aim of the present study is to report the neuropsychological evaluation of a large sample of SMBA patients in order to contribute towards the definition of these patient’s cognitive profile.

Namely, in our study we retrospectively analysed the results of neuropsychological tests of a large population of patients with SBMA evaluated from 2013 to 2015, attending as outpatients at the Neuromuscular Centre of the University of Padova (Italy). In detail, the archived data consisted of a routinely administered brief neuropsychological battery aimed to a fast evaluation of short- and long-term memory, linguistic abilities and executive functioning (for details please see methodological section). The tasks’ choice had a clinical finality and was aimed to assess the integrity of the cognitive functions in order to eventually consider hinderances to an adequate communication with the doctor, or that could undermine patients’ compliance in the home management of care.

Furthermore, since previous studies suggested that AR mutation-related androgen resistance and testosterone levels could impact on cognition^20^, we also investigated the relationship of cognitive performance with CAG-repeat expansion and the degree of androgen insensitivity in these patients.

## Results

### Subjects

Demographic indexes of controls and SBMA patients, and patients’ main clinical features are summarized in Table 1. As shown in this table, t-tests did not reveal any difference between patients and controls mean age and education.

**Table 1:** Demographic indexes and main clinical features of the studied populations.

	Patients (n = 64)		Controls (n = 78)		t-test	
	Mean	SD	Mean	SD	T(df)	p-value
Age	57.27	10.47	56.15	14.08	0.52 (140)	0.601
Education	11.09	4.23	11.12	4.22	−0.03 (140)	0.976
AR-CAG repeats	46.06	2.62	—	—		
ALSFRS-R	41.90	3.71	—	—		
6MWT	357.95	126.75	—	—		
ASI	138.71	100.53	—	—		

Age and education are expressed in years; AR-CAG: androgen receptor-cytosine adenine guanine; ALSFRS-R: amyotrophic lateral sclerosis functional rating scale - revised; 6MWT: 6-minute walking test, expressed in meters; ASI: Androgen Sensitivity Index, expressed in U * nmol/L^2^; SD: standard deviation.

### Neuropsychological findings

ANCOVA results showed no statistically significant difference between groups in all the examined test performance, except for the Babcock Story Recall Test score, in which patients performed better than control participants, with no relevant influence of age and education. Education level was instead a statistically significant covariate for all the other measures, while participants’ age was found statistically significant for the TMT B-A and the DSf scores only. Results are illustrated in Tables 2 and 3.

**Table 2:** Neuropsychological results.

	Patients (n = 64)		Controls (n = 78)	
	Mean	SD	Mean	SD
PM	13.54	2.58	12.35	2.57
FAS	38.73	10.34	35.79	11.49
TMT B-A	−3.88	0.52	−3.67	0.80
DSf	5.83	1.23	5.93	1.21
DSb	4.41	1.14	4.10	1.00

PM: Prose Memory test; FAS: phonemic fluency test; TMT B-A: trial making test, log-transformed difference between B and A subtests; DSf: digit span forwards; DSb: digit span backwards; SD: standard deviation.

**Table 3:** ANCOVA analyses.

Response	Predictor	SS	Df	F	Pr (>F)	Partial eta^2^	Cohen’s d^†^
PM	Age	15.40	1	2.42	0.123	0.02	0.50
	Education	13.81	1	2.17	0.143	0.02	
	Group	50.42	1	7.91	**0.006**	**0.06	
	Residuals	802.78	126				
FAS	Age	21.39	1	0.19	0.660	0.00	0.28
	Education	1321.87	1	12.00	**<0.001**	***0.08	
	Group	293.70	1	2.67	0.105	0.02	
	Residuals	14544.48	132				
TMT B-A	Age	1.85	1	4.70	**0.032**	*0.04	0.24
	Education	3.70	1	9.38	**0.003**	**0.07	
	Group	0.72	1	1.83	0.179	0.01	
	Residuals	47.70	121				
DSf	Age	1.23	1	5.97	**0.016**	*0.04	0.07
	Education	3.55	1	17.19	**<0.001**	***0.12	
	Group	0.03	1	0.13	0.715	<0.01	
	Residuals	27.23	132				
DSb	Age	0.60	1	2.50	**0.117**	0.02	0.34
	Education	2.39	1	9.94	**0.002**	**0.08	
	Group	0.84	1	3.50	0.064	0.03	
	Residuals	26.96	112				

^†^Computed on adjusted scores; Significance levels: *p < 0.05; **p < 0.01; ***p < 0.001.

PM: Prose Memory test; FAS: phonemic fluency test; TMT B-A: trial making test, log-transformed difference between B and A subtests; DSf: digit span forwards; DSb: digit span backwards; SS: sum of squares; Df: degrees of freedom.

### Relationship between neurological and cognitive measures

The regression analyses within the patients’ group, reported in Table 4, did not show support for any hypothesized effects of CAG-repeat expression, the ASI index, or their interaction, on patients’ cognitive performance. All the models showed low R^2^ and evidence ratios against the covariate-only models lesser than 1, meaning that introducing the additional dependent variables reduced the models’ quality of fit.

**Table 4:** Within-patient regression analyses.

Response		Age	Education	ASI	CAG repeat	Interaction ASI × CAG	R^2^	Evidence ratio
PM	Estimate	0.03	0.124	−0.108	−0.053	0.002	0.055	0.26
	Std. Error	0.047	0.085	0.073	0.259	0.002		
	t	0.637	1.463	−1.481	−0.206	1.463		
	p-value	0.527	0.149	0.144	0.838	0.149		
FAS	Estimate	0.003	0.566	−0.435	−0.138	0.01	0.162	0.65
	Std. Error	0.161	0.316	0.271	0.977	0.006		
	t	0.016	1.793	−1.602	−0.141	1.585		
	p-value	0.987	0.078	0.115	0.888	0.119		
TMT B-A	Estimate	−0.017	0.029	−0.022	−0.065	0	0.246	0.12
	Std. Error	0.008	0.015	0.013	0.047	0		
	t	−2.237	1.931	−1.692	−1.393	1.703		
	p-value	0.029*	0.059	0.096	0.169	0.094		
DSf	Estimate	−0.003	0.021	−0.011	−0.029	0	0.308	0.04
	Std. Error	0.007	0.014	0.012	0.043	0		
	t	−0.43	1.523	−0.939	−0.677	0.947		
	p-value	0.667	0.128	0.348	0.498	0.344		
DSb	Estimate	−0.001	0.009	−0.015	−0.028	0	0.134	0.06
	Std. Error	0.008	0.016	0.014	0.05	0		
	t	−0.154	0.573	−1.109	−0.573	1.102		
	p-value	0.877	0.567	0.267	0.567	0.27		

Significance levels: *p < 0.05.

CAG repeats: cytosine-adenine-guanine repeats.

## Discussion

No frank cognitive impairment was found in our retrospective study on 64 patients with SBMA, as deduced from the scores of patients on neuropsychological tests, compared with those of healthy male subjects. Surprisingly, patients showed better performance on the Prose Memory test score^21^.

In detail, no significant lower scores were found in the Digit Span forwards test^21^ (used to evaluate short-term memory ability for non-logically organized verbal material, i.e. numbers), in the Digit Span backwards test^21,22^ (similarly to Digit Span forwards test, it is used to evaluate the short-term memory, jointly to the executive functioning, as it is based on internal manipulation of mnesic representations of information in the absence of external cues), in the Prose Memory test total score^21^ (used to evaluate memory for logically organized material), in the Phonemic Fluency test^23^ (used to evaluate both linguistic skills and executive functions, as it requires high order functioning to organize internal information, i.e. first-letter driven words’ choice, in a non-habitual manner)^24^ and on the difference between TMT B and TMT A sub-tests^25^ (index used to evaluate executive functions in terms of set-shifting skills)^26^.

Our results are apparently in contrast with some previous empirical studies, in some of which cognitive defects have been reported in SBMA population^13–16^. Namely, Guidetti and colleagues^13^, found abnormal scores mainly on tests evaluating long-term memory and executive functions. In that study, a broad neuropsychological battery was proposed, including Digit Span forwards, the Prose Memory test and the Phonemic Fluency test – which were as well administered to our patients. Out of the 5 affected patients (3 clinical and 2 preclinical) the authors evaluated, 4 revealed abnormal performance to at least one of the proposed neuropsychological tests. Of note, altered tests scores were different for each patient, hence subtending different cognitive functions resulting impaired. In detail, one clinical patient failed also in Prose Memory test and one preclinical patient failed in Phonemic Fluency test.

In a further study, Soukup and affiliates^14^, found that their group of 20 patients with SBMA underperformed in many tests of a detailed neuropsychological test battery, which have in common with ours the Digit Span forwards and backwards tests. A most pronounced impairment in the domains of short- and long-term memory, language and attentional control, has been found. However, impairment appeared as mostly subclinically expressed.

Kasper and colleagues^15^ found that 20 patients with SBMA they evaluated performed significantly worse than healthy controls in the mnesic, executive and attentional control domains. Differently from our results, they found this low performance scores in Digit Span forwards and backwards tasks, and in Phonemic Fluency test. However, similarly to what was found in Soukup and colleagues’ study, the impairments resulted as subclinical and not relevant to the patients’ everyday functioning. Yang and colleagues^16^ found impaired clinical/subclinical performance on 10 patients in the short- and long-term memory and/or in the executive functions, for the evaluation of which they have used, as in our study, the TMT and the Phonemic Verbal Fluency test.

Finally, in our previous study^17^ we found in 20 patients affected by SBMA a significant impaired performance in Theory of Mind (ToM)^27^ abilities, as detected by Faux Pas Task^28^, a measure employed to assess cognitive aspects of ToM in terms of understanding other’s thoughts and intentions, an ability considered to be interconnected, but not overlapping, to executive functioning^29^. Affective aspects of ToM, assessed with Reading the Eyes in the mind test^30^, resulted spared.

As a whole, the picture that emerges from an overview of previous studies is the possible mild cognitive impairment in these patients at mnesic, executive and attentional control levels, which however rarely reaches appreciable clinical threshold. Furthermore, the deficits appear to be heterogeneous, and the same tests do not always give significantly altered results in the different samples of these studies. This perspective suggests that probably there is no peculiar or prototypical compromised profile in these patients, as in the case, conversely, of Amyotrophic Lateral Sclerosis (ALS), the other known syndrome belonging to the family of motor neuron diseases (for a review see Pinto-Grau, Hardiman, & Pender, 2018)^31^, in which is it well established that cognitive impairment, when present, is primarily manifested in the patients’ failure at Phonemic Fluency test^32^.

The inconsistency between the absence of cognitive alterations that we find in our wide sample and the presence of alterations, of various degrees and nature, found in other studies with smaller samples, may be interpreted in the light of the above-mentioned heterogeneity of cognitive defects emerging from the comparison of previous studies. If cognitive defects were specific for each patient and extremely mild, they could have flattened by applying statistical analyses on a larger sample. In other words, our wide sample may have diluted the specific alterations of the patients we assessed, especially if the impairment was at subclinical level and different from one patient to another.

This interpretation nicely fits with the phenomenon of the so called somatic mosaicism, characterizing this pathology^33^. It implies that the number of CAG-repeats is not constant in every cell of an individual, but it may vary across tissues, including the cerebral ones; such an instability is typical of other neuromuscular diseases (for a review see Laing, 2012)^34^.

The mutant androgen receptor protein, peculiar of SBMA disease, cleaved by protein caspase‐3 at Asp146, generates a further cleaved product that seems to be toxic to cells^35^, likely resulting in cell degeneration. Considering the phenomenon of somatic mosaicism, it follows that mutant androgen receptors’ protein and their toxic agglomerates could be differently expressed in patients’ cells, also in respect to different neural tissues and areas.

It is hence possible, indeed, that neuronal alterations leading to a cognitive defect are only occasionally present in SBMA patients, and could involve different cognitive functions underpinned by different neural networks, although they occur mainly at frontal or fronto-temporal levels, as indeed highlighted by previous neuropsychological, neuroimaging, and electrophysiological studies^4–17^. This interpretation could be in line with the sporadic cases of comorbidity with fronto-temporal dementia and SBMA reported in literature^18,19^.

The second result for which an in-depth possible theoretical interpretation is needed is the patients’ better performance at Prose Memory test in respect to control subjects. This result has never been described previously in this disease and, in general, is extremely infrequent in neurological disorders (out of the rare “paradoxical functional facilitation”; see Kapur in 1998)^36^. The significantly greater score in this test could be firstly interpreted considering patients’ hormonal peculiarities: their androgen receptors insensitivity, due to their genetic mutation, implies indeed a lower uptake of this androgen hormone in all body tissues, resulting also in typical SBMA symptoms such as gynecomastia or hypogonadism. It has been well established that testosterone mediates also several human (and animal) behaviours and attitudes, besides morpho-functional sexual dimorphisms. A number of previous researches demonstrate that women have a better memory for emotional information than men^20,37^, and that emotional information is rated as more arousing than in males^38^. This interpretation could be also in line with the Empathizing-Systemizing (ES) theory of Baron-Cohen^27^, supporting evolutionary roots for the phenomenon that females show, on average, a stronger drive to empathize than males. The role of testosterone is at the basis of these theoretical and empirical contributions distinguishing males and females in their emotional processes and empathizing^39^: the higher the testosterone level, the lower the capacity to be involved in an affective/empathic relation. For instance, a single administration of testosterone in female subjects leads to an impairment in the ability to infer emotions, intentions, and mental states of others^40^. Compared to other memory tasks in neuropsychological assessment field, Prose Memory test^21^ has a significant affective connotation. Indeed, it consists in a story with a strong emotional content, likely elicitating empathic feelings towards the victims reported in the task’s text. Hence, even if it is not a measure addressed to assess emotional/empathic processing, its clear emotional content cannot be neglected in the light of our unexpected results. This first testosterone-related interpretation of our results is therefore linked to the fact that the lower absorption of testosterone in the brain tissues of these patients (as observed by Tanaka and colleagues in 1992 in post-mortem studies^33^, androgen mutant receptors in SBMA are also present in subcortical nuclei such has amygdala, which has a pivotal role in affective empathy) may have facilitated the emotional encoding and/or empathic processing. This finding could be conceived also in line with our previous findings highlighting a dissociation between impaired cognitive ToM abilities and spared affective ToM abilities in these patients^17^.

A second perspective related to the best performance linked to the Prose Memory test is related to the possible presence of subclinical depression in these patients. As it is known, the presence of severe depression is an important factor in the alteration of memory, attention and executive functions^41^. However, SBMA disease does not imply a severe physical impairment, so that a major depression is not a typical reaction in the great majority of cases. However, since it is a disease with manifest clinical consequences, even if slight, we cannot rule out the presence of a widespread form of subclinical depression. This supposed low mood may have facilitated the recall of information with negative emotional content, in line with the empirically proven mood-dependant state^42^. According to this second interpretation, the possible deflected mood of these patients may have led to a better performance in the recall of a story rich in elements with negative emotional value, as it is consistent with their possible coping towards the disease. Unfortunately, as patients’ mood was not evaluated with specific questionnaires, this interpretative argument remains elusive.

Finally, conversely with our expectations, ASI index, testosterone-level related, CAG-repeat expression, and their interaction did not reveal any significant association with neuropsychological tests administered to our patients, including the Prose Memory test^21^, as showed by our regression analyses.

Although it is reasonable to hypothesize associations between the genetic, hormonal and clinical profiles of patients and their performance in neuropsychological tasks, the absence of such association should not be surprising. Some studies before ours, both based on the neuropsychological profile and the morpho-functional brain profile of these patients, did not confirm their hypothesis on the presence of associations between their results and the genetic, clinical and hormonal variables of the patients^15,43^. The somatic mosaicism phenomenon^33,34^ could imply that such parameters, directly or indirectly related to genetic expression in the body districts, could not be associated to their expression in the brain tissues, hence to neuropsychological performance.

Despite our intriguing results, our study is plagued by several limitations. First of all, it has the disadvantages of retrospective studies, such as the lack of planning of a straightforward, preliminary hypothesis in the choice of the tests. Consequently, neuropsychological tests we proposed to the patients do not adequately cover all cognitive functions, nor allow a fine-grained analysis of functions we investigated, i.e. short- and long-term memory, language and executive functions. For example, a replication of one of the previous studies on the cognitive profile of these patients, using the same tests’ battery but on our larger sample, would have been extremely useful as a scientific contribution to disambiguate the conflicting results we found. The lack of a concomitant evaluation of the mood, to be investigated as possible moderator or mediator of our results to neuropsychological tests, represents another of the limits of our study.

Notwithstanding these considerations, given the rarity of the disease and the paucity of published information on these patients’ cognitive profile, our report on such a large sample of people affected by SMBA represents an interesting contribution, with high statistical power, from where further fine-grained research question can develop. Indeed, to the best of our knowledge, this is the neuropsychological study with the highest number of SBMA participants in the current scientific panorama. As a whole, our findings can contribute to patients’ clinical care and add a piece in the discovering puzzle of the not still completely understood SBMA syndrome.

Future prospective studies based on more detailed neuropsychological battery and implemented by electrophysiological/neuroimaging investigations are warranted. Moreover, a promising direction would be the investigation of the emotional/empathic features of these patients, as well as an evaluation of mood state to exclude interference with cognitive performance.

## Methods

### Participants

We retrospectively considered all neuropsychological evaluations consecutively collected in 105 SBMA patients attending the Neuromuscular Center of the University of Padova from 2013 to 2015. All of them had genetically confirmed diagnosis of SBMA and their medical history was exhaustive about clinical data, concomitant diseases and pharmacological treatment. Inclusion criterion was being of Italian mother tongue and exclusion criteria were concomitant neurological diagnosis at risk to affect cognition, such as previous stroke, psychiatric conditions, depressive symptomatology, or current use of high-dose psychoactive medications. Sixty-four patients fulfilled inclusion and exclusion criteria and were considered eligible for this study. The cognitive examination was carried out by a neuropsychologist with wide experience in the field of neuromuscular disorders (S.M.), during the same day of the neurological examination performed by a senior neurologist (G.S.). Hormonal profile including serum concentrations of total testosterone, luteinizing hormone (LH) and follicle-stimulating hormone (FSH) was collected. Testosterone values were multiplied for LH to determine the Androgen Sensitivity Index (ASI) (normal values < 138 U * nmol/*L*^2^)^44^. Genetic DNA was extracted from peripheral blood using standard protocols. CAG-repeats were amplified by polymerase chain reaction as elsewhere described^45^. Repeat fragment sizing was performed on an ABI Prism 3700 DNA Sequencer (Applied Biosystems, Foster City, CA, USA). The specific length of CAG-repeats was confirmed via Sanger sequencing. Total testosterone levels were tested on blood according to standard protocols.

Clinical assessment consisted of a full neurological exam completed by a functional evaluation using the revised Amyotrophic Lateral Sclerosis Functional Rating Scale (ALSFRS-R)^46^ since no other scales specifically adapted to SBMA, i.e. Spinal and Bulbar Muscular Atrophy Functional Rating Scale (SBMAFRS)^47^, were available at the study time period. ALSFRS-R is a scale considering motor, bulbar and respiratory functions approved to describe functional status of patients with amyotrophic lateral sclerosis (ALS)^46^. Because the scale items are applicable to SBMA, the ALSFRS-R has been used to monitor disability progression in SBMA patients able to walk with or without aid^48^. Functional status was also assessed using the 6 Minute Walk Test (6MWT). The 6MWT is performed by calculating the distance covered by the patient walking for 6 minutes along a flat corridor of 25 meters of length. It is usually considered the most reliable biomarker of motor impairment in SBMA and presents a decline of about 8% over one-year observation time^49^.

Seventy-eight male healthy controls who fulfilled the same criteria were recruited from local volunteer associations and underwent the same neuropsychological battery. All participants signed an informed consent at the time of the consultation to make available their anonymous data for research purposes. Our retrospective study was approved by the psychological research ethical committee, Area 17, of the University of Padova, in line with the declaration of Helsinki.

### Neuropsychological assessment

The neuropsychological battery proposed to patients and healthy controls was composed by standardized neuropsychological instruments taken from Spinner and Tognoni Battery^21^. The evaluation, lasting about 30 minutes, was composed by tests selected for their simplicity and speed of execution, as our patients were recruited in an outpatient modality.

In detail, we employed:

- *Phonemic Fluency Test* (FAS)^23^: It is considered a task able to assess both linguistic skills and executive functions, as it is based on the ability to categorize words in a non-habitual way, thus implying planning and strategy formation, typical of high order functioning. The subject has to say as many words as possible in one-minute time, starting with a given letter of the alphabet, i.e. F, A and S. The score used was relative to the sum of all the words produced by the participant in three minutes.
- *Trial Making Test* (TMT)^25^: It is composed by subtests A and B. In the A subtest, the subject has to connect with a line the numbers from 1 to 25 in the shortest time possible, and it is employed to assess attentive abilities at very basic level. In the B subtest the participant has to connect with a line numbers and letters, alternating the two different kind of symbols and following both the numerical and the alphabetical order; it implies both basic attentive abilities and executive functioning mainly in terms of set-shifting. For both tests the score represents the time needed to complete the tasks. The difference between the B and A (TMT B-A) was calculated and employed in statistical analysis in order to observe the pure effect of executive functioning.
- *Prose Memory test* (PM)^21^: Also known as Babcock Memory test, it is a task aimed to evaluate memory function in terms of recall of logically-organized verbal information. It consists in a brief story read by the examiner to the participant. The text narrates about a flood that hit a small city, causing the destruction of many buildings and the death of some people despite the rescues’ attempts. Immediately following the first reading, the subject is asked to recall everything he remembers about the story. After the first recall phase, the examiner reads again the story. The recall following the second reading is asked after 10 minutes in which the subject makes non-interfering activity with mnesic encoding. Each main element of the story correctly recalled has a specific score. The global score of the task is obtained by the sum of the immediate and the delayed recall scores.
- *Forwards Digit Span* (DSf)^21^: It is a widely employed task to assess short-term verbal memory of non-logically organized material (casual numbers). In this task the participant had to memorize increasing series of digits. The task ends when the participant is no longer able to correctly recall two consecutive numerical strings of the same length.
- *Backwards Digit Span* (DSb)^21^: Similarly to DSf, the participant was asked to repeat the digits read by the examiner, but in the reverse order. Also in this case, the task ends when the participant is no longer able to correctly repeat the inverse order of the numbers of two consecutive numerical strings of the same length. As this task requires maintaining the items, but also mentally manipulating them engaging additional resources, it is considered a reliable measure both of short-term memory and executive functions abilities.

Since the neuropsychological evaluation was almost always made during the first visit, the motor deficits were very mild. Hence, no adjustments were made in the time-dependent tests based on verbal speech because none of the patients showed evidence of bulbar disabilities that might had affected their neuropsychological performance, as indicated by the items of the ALSFRS-R relative to the verbal domain (M = 3.56; SD = 0.50). Even if TMT B-A index it is not altered by motor impairment, fine motor impairment at upper limbs was not present in these patients (M = 3.65; SD = 0.43. See also Table 1 for ALSFRS-R total score).

### Data analysis

Analyses were carried out using R software version 3.3.1^50^. Univariate ANCOVAs were performed to determine a statistically significant difference between patients and control participants on PM, FAS, TMT B-A, DSf, and DSb scores. Multiple linear regressions were performed to investigate the hypothesized relationships between genetic expression and ASI index and patient’s cognitive performance. Evidence ratios^51^ and R^2^ were employed to evaluate the models’ support to the hypotheses. In both analyses, in respect of the count nature of DSf and DSb scores, Poisson regressions were employed to estimate such models. Logarithmic transformation was applied to TMT B-A scores to reduce their strong skewedness, furthermore the transformed scores were sign reversed so that higher scores would represent better performance, analogously to the other measures. As there is some evidence that educational level and aging can influence performance on similar tasks^52^, all analyses used age and education as covariates. The compliance of the data to ANCOVA and regression assumptions were graphically investigated and raised no major concern. Finally, Pearson correlation coefficients were employed to assess the associations between cognitive performance and measures of physical impairment (ALSFRS-R and 6MWT), and between CAG-repeat expression and ASI index. No relevant correlations were found between these variables. The sample size for all the analyses was n = 64 in the patients group and n = 78 in the control group.

## Data Availability

The datasets generated and/or analyzed during the current study are available from the corresponding authors on specific request.
